# Nanoparticle Encapsidation of *Flock House Virus* by Auto Assembly of *Tobacco Mosaic Virus* Coat Protein

**DOI:** 10.3390/ijms151018540

**Published:** 2014-10-14

**Authors:** Payal D. Maharaj, Jyothi K. Mallajosyula, Gloria Lee, Phillip Thi, Yiyang Zhou, Christopher M. Kearney, Alison A. McCormick

**Affiliations:** 1 Department of Biological and Pharmaceutical Sciences, Touro University, Vallejo, CA 94594, USA; E-Mails: pamahara@utmb.edu (P.D.M.); mjyothik@gmail.com (J.K.M.); gloria.lee@tu.edu (G.L.); philip.thi@tu.edu (P.T.); 2 Department of Biology, Biomedical Studies Program, Baylor University, Waco, TX 76706, USA; E-Mails: Yiyang_Zhou@baylor.edu (Y.Z.); Chris_Kearney@baylor.edu (C.M.K.)

**Keywords:** *Flock House virus*, *Tobacco Mosaic virus*, encapsidation, self-assembly, nanoparticle, transgene expression, viral vector

## Abstract

*Tobacco Mosaic virus* (TMV) coat protein is well known for its ability to self-assemble into supramolecular nanoparticles, either as protein discs or as rods originating from the ~300 bp genomic RNA origin-of-assembly (OA). We have utilized TMV self-assembly characteristics to create a novel *Flock House virus* (FHV) RNA nanoparticle. FHV encodes a viral polymerase supporting autonomous replication of the FHV genome, which makes it an attractive candidate for viral transgene expression studies and targeted RNA delivery into host cells. However, FHV viral genome size is strictly limited by native FHV capsid. To determine if this packaging restriction could be eliminated, FHV was adapted to express enhanced green fluorescent protein (GFP), to allow for monitoring of functional FHV RNA activity. Then TMV OA was introduced in six 3' insertion sites, with only site one supporting functional FHV GFP expression. To create nanoparticles, FHV GFP-OA modified genomic RNA was mixed *in vitro* with TMV coat protein and monitored for encapsidation by agarose electrophoresis and electron microscopy. The production of TMV-like rod shaped nanoparticles indicated that modified FHV RNA can be encapsidated by purified TMV coat protein by self-assembly. This is the first demonstration of replication-independent packaging of the FHV genome by protein self-assembly.

## 1. Introduction

Macromolecular assembly of virus capsid proteins has been used to create new formulations of virus-like particles (VLPs) and virus particles. One of the earliest studied self-assembly processes is that of *Tobacco Mosaic virus* (TMV); Fraenkel-Conrat *et al.* first demonstrated that infectious TMV could be reconstituted *in vitro* from purified RNA and TMV coat protein under specific physiological conditions [1]. Further studies defined coat protein self-assembly properties [2], and characterization of a small RNA sequence within the TMV RNA that independently directs encapsidation [3]. This sequence was then used to direct encapsidation of non-native RNA content, of either a hybrid TMV RNA segment including a non-native 3' end [2], or a small gene coding sequence [4] that was successfully tested for co-translational protein expression. These original studies described the useful properties of the TMV origin-of-assembly (OA) in directing macromolecular self assembly, but did not further explore the use of the interaction between the OA and TMV coat protein in creating a functional replicating RNA. In our previous studies, we extended the functionality of OA directed self assembly by TMV coat encapsidation of a modified *Semliki-Forest virus* (SFV) RNA. SFV and TMV are distantly related alpha virus family members, and share certain similarities in the life cycle that made it more likely to create a functional encapsidated particle, capable of withstanding insertion of the OA without disrupting SFV function. Expression of a reporter transgene *in vitro* suggested successful co-translational disassembly, and immunization and immune reactivity to the encapsidated transgene confirmed SFV function *in vivo* [5]. Although these studies demonstrated that a novel virus composition could be created by TMV coat self assembly, there were limitations in the use of SFV encapsidated RNA, including a large RNA genome size that was unstable with the introduction of transgenes of interest, an inability to move SFV into *in vivo* RNA expression systems, and induction of apoptosis in cells exposed to SFV that might limit immune responses to encoded transgenes. Since the ultimate use of the trans-encapsidated RNA was for vaccine development, we have explored the TMV coat protein encapsidation of RNA1 from *Flock House virus* (FHV) in order to overcome some of these limitations. Although the replication and packaging of FHV is greatly divergent from alphavirus members like SFV and TMV [6,7], its high level replication [8], small genome size, simple organization [9], and suppression of apoptosis [10] were attractive characteristics in developing a more robust encapsidated RNA system.

*Flock House virus* belongs to the Nodaviridae family and the *Alphanodavirus* genus, and was first isolated from the grass grub *Costelytra zealandica* (Coleoptera) in New Zealand [11]. FHV is a unique insect virus in that it is able to cross multiple kingdom barriers and can replicate in plants [12,13], insects [14,15] and yeast [16]. FHV has a simple genome organization composed of two positive-sense, single-stranded RNAs packaged by a single capsid into a nonenveloped icosahedral virion [1]. RNA1 is 3.1 kb in length and encodes the autonomous viral RNA-dependent RNA polymerase (RdRp, protein A; 112 kDa). During FHV replication, a subgenomic RNA3 (0.4 kb) is also synthesized which encodes two proteins, B1 and B2 [17]. The function of translated B1 protein is poorly defined, but may be important for maintenance of RNA replication [12], whereas protein B2 is responsible for suppressing Dicer-mediated RNA silencing [18]. Genomic RNA2 (1.4 kb) encodes the viral capsid protein precursor, CP-α (43 kDa), that is later cleaved into 40 kDa (β) and 4 kDa (γ) fragments after provirion assembly [19,20].

The autonomous ability of the FHV RNA1 to replicate and the robust intracellular genome synthesis and protein expression directed by subgenomic promoters makes FHV an ideal candidate for amplifying heterologous sequences. The first construction of nodavirus RNA1 and RNA2 transcription plasmids in the T7-promoter driven constructs yielded *in vitro* transcribed RNA that produced infectious virions in Drosophila cells [21]. Further work with nodaviruses led to the development of FHV cDNA based replicon systems that successfully generated FHV viral proteins *in vitro* in mammalian cells [22]. A yeast DNA system was also developed where wild-type FHV RNA1 was expressed *in vivo* and this system was successfully utilized to express green fluorescent protein (GFP) [23]. Vectors have also been developed from the cDNA clones of both FHV RNA1 and RNA2 for the expression of reporter genes such as GFP in Drosophila cells, yeast and mosquitoes [9,15,16,24].

Successful packaging of viral vectors expressing heterologous genes becomes limited as native capsid or envelope proteins either have to be provided in trans or the vector has to be expressed in a cell line that constitutively expresses viral structural proteins [25]. Additionally, most viruses have evolved stringent genome sizes so heterologous gene insertions are not well tolerated and result in poor packaging of *in vitro* or *in vivo* synthesized chimeric forms of viral RNA by native capsid [26]. This is especially true of very small viruses, which have co-adapted genome size and packaging characteristics. Packaging of FHV RNA1 transcript by the CP-α is highly specific and is dependent on a physical interaction between protein A and the CP-α occurring at the sites of replication on the mitochondria [27]. An RNA-binding motif called the Arginine Rich Motif (ARM) that has been identified in several viral CPs and FHV RNA1 has been shown to contribute to the specificity of viral genome packaging [27]. Origin-of-assembly (OA) sequences have also been associated with viral genome assembly and packaging; however presence of either the ARM or the OA does not always result in successful *in vitro* encapsidations [26]. Therefore, specificity of viral encapsidation signals can allow for packaging of viral vectors in non-native packaging structures as suggested by Zhong *et al.* [28] and confirmed by others [5].

In order to overcome packaging limits by FHV native capsid and to maintain FHV RNA1 replication ability, a synthetic method for encapsidating the FHV RNA1 vector with foreign viral coat protein was investigated. The TMV OA is a short ~300 nucleotide sequence that is recognized by the TMV coat protein (CP) and allows for the initiation of virion assembly [2,28]. This supramolecular self-assembly requires a specific interaction between a defined TMV CP aggregate and the TMV OA, where there is a sequential addition of smaller CP aggregates on a lengthening helical rod starting at a loop of RNA around the OA [2]. Following the methodology in that study, the present study had two objectives (1) to develop FHV as a transgenic expression vector and (2) to demonstrate that OA containing FHV RNA can be encapsidated into nanoparticles by TMV CP (independent of sequence identity or length) by coat protein self-assembly. The development of the *Flock House virus* vector for expression of GFP, its utility as a protein expression vector and trans-encapsidation with TMV CP is demonstrated.

## 2. Results and Discussion

### 2.1. Synthesis and Organization of FHV-GFP Expression Clones

**Figure 1:**
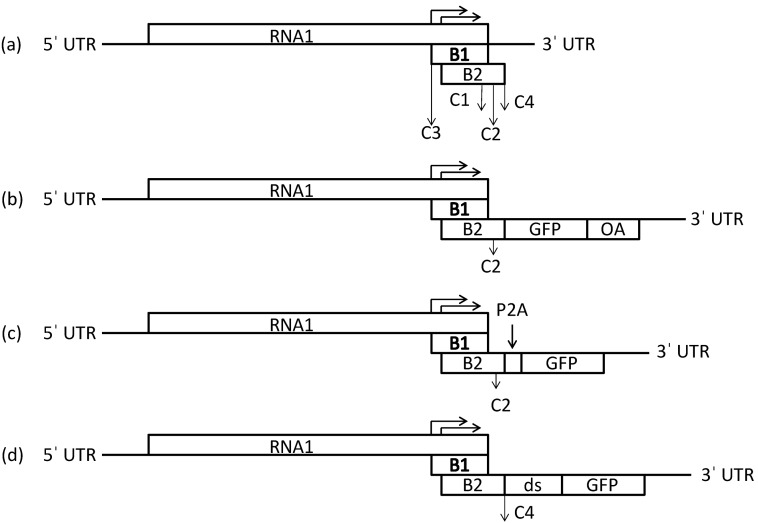
Schematic representation of the *Flock House virus* RNA1 vector constructs. (**a**) FHV RNA1 genome with subgenomic proteins, B1 and B2. Arrow positions correspondto insertion sites that were engineered into the FHV RNA1 clone: C1- 3034 bp, C2- 3037 bp, C3- 2731 bp, C4- 3055 bp; (**b**) FHV-C2-GFP-P2A; (**c**) FHV-C4-GFP-ds where ds1 is 2518–3055 bp and ds2 is 2518–3107 bp of FHV RNA1 were cloned into FHV-C4-GFP respectively; and (**d**) FHV-C4-GFP-RNA2 where 509–868 bp from FHV RNA2 was cloned into FHV-C4-GFP.

FHV was adapted for GFP expression, by insertion of GFP under the control of B1 or B2 subgenomic promoters, or by duplication of the 3' FHV RNA (ds) or as enhanced by insertion of an RNA2 promoter as shown schematically in Figure 1. The 2A peptide (P2A) clones introduced a protein cleavage site to enhance GFP translation.

### 2.2. Fluorescence of FHV-GFP Clones in Mammalian Cells

**Figure 2:**
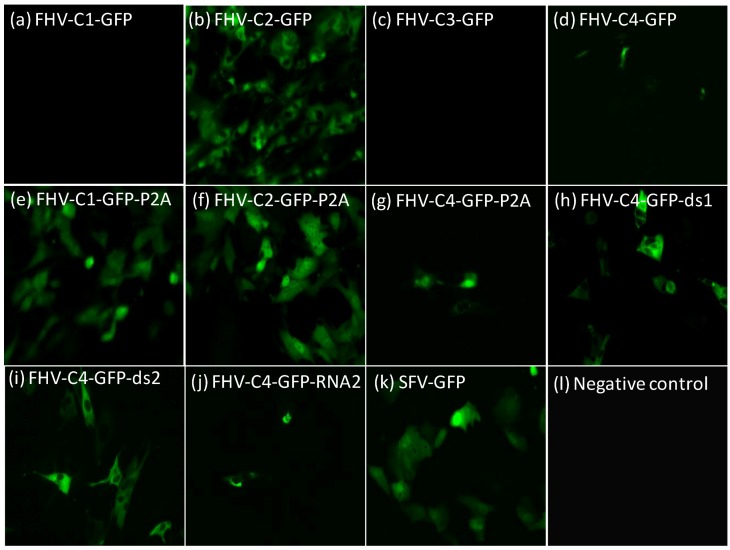
Fluorescence images of FHV RNA1 GFP plasmid constructs. BHK-21 cells were transfected with 2 µg of *in vitro* transcribed FHV RNA with DMRIE-C transfection reagent. Transfected cells were incubated at 37 °C for 4 h after which fresh growth media was added and cells transferred to 28 °C. Images were taken at 48 h post-transfection with a Nikon Eclipse TS-100 microscope and NIS Elements BR 4.11.00 imaging software. All images are at a 400× magnification. (**a**) FHV-C1-GFP; (**b**) FHV-C2-GFP; (**c**) FHV-C3-GFP; (**d**) FHV-C4-GFP; (**e**) FHV-C1-GFP-P2A; (**f**) FHV-C2-GFP-P2A; (**g**) FHV-C4-GFP-P2A; (**h**) FHV-C4-GFP-ds1; (**i**) FHV-C4-GFP-ds2; (**j**) FHV-C4-GFP-RNA2; (**k**) SFV-GFP-OA; and (**l**) non-transfected cells.

After constructed plasmids were verified by sequencing, RNAs were transcribed and transfected into BHK-21 cells, and monitored for their ability to sustain GFP expression. Fluorescence was not observed with FHV-C1-GFP at 48 h (Figure 2a) whereas strongest fluorescence was observed with FHV-C2-GFP at 48 h (Figure 2b). Similar to FHV-C1-GFP, clone FHV-C3-GFP did not yield detectable fluorescence at 48 h (Figure 2c). Weak expression was noted with FHV-C4-GFP at 48 h (Figure 2d). Insertion of the P2A cleavage sequence considerably improved GFP expression for FHV-C1-GFP-P2A (Figure 2e) in comparison with the parental FHV-C1-GFP. Expression of GFP was maintained in the FHV-C2-GFP-P2A clone (Figure 2f). In contrast, GFP expression in the FHV-C4-GFP-P2A (Figure 2g) was lower in comparison with FHV-C4-GFP.

Expression of GFP with FHV-C4-GFP greatly improved with the addition of the double sub-genomics, ds1 (Figure 2h) and ds2 (Figure 2i) at 48 h. GFP expression with the FHV-C4-GFP-RNA2 (Figure 2j) clone improved slightly in comparison with the parental FHV-C4-GFP clone at 48 h. A previously developed SFV-GFP clone was used as a positive fluorescence control (Figure 2k).

### 2.3. GFP Expression Kinetics

**Figure 3:**
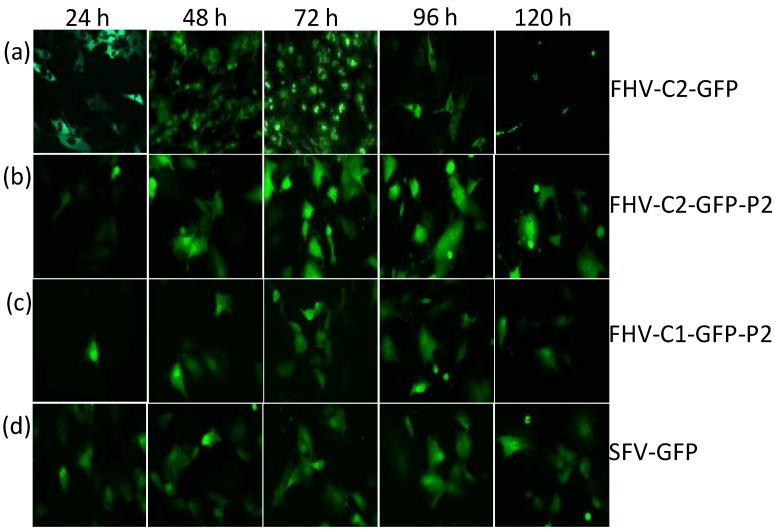
Time course assay observing daily fluorescence of different FHV GFP clones over a period of 120 h. BHK-21 cells were exposed to 2 µg of FHV RNA via liposome-mediated transfection, incubated at 28 °C and observed for fluorescence daily. All cell images are at a 400× magnification: (**a**) FHV-C2-GFP; (**b**) FHV-C1-GFP-P2A; (**c**) FHV-C2-GFP-P2A; and (**d**) FHV-C4-GFP-P2A and SFV-GFP. Images from expression vectors that had undetectable to low levels of fluorescence were not included in this figure.

In order to assess GFP expression and accumulation levels over time, a time course assay was set up over a period of five days and cells were visualized by fluorescence microscopy (Figure 3). Fluorescence was observed within 24 h with FHV-C2-GFP with highest fluorescence at 72 h that waned over time (Figure 3a). Addition of P2A to FHV-C2-GFP did not improve expression, however, fewer cells were fluorescing as compared to the parental clone; expression kinetics remained the same as FHV-C2-GFP (Figure 3b). No fluorescence was observed with FHV-C1-GFP at any timepoint (data not shown), whereas FHV-C1-GFP-P2A showed weak fluorescence at 24 h that peaked at 72 h and diminished over time (Figure 3c). Expression of GFP with SFV-GFP was observed at 24 h and maintained until 120 h (Figure 3d). Fluorescence expression kinetics of FHV-C1-GFP, FHV-C3-GFP, FHV-C4-GFP, FHV-C4-GFP-P2A, FHV-C4-GFP-ds1, FHV-GFP-ds2 and FHV-C4-GFP-RNA2 followed the general pattern of FHV-C2-GFP, but were not included in this figure since they were low expressing clones.

### 2.4. Efficiency of P2A Cleavage

**Figure 4:**
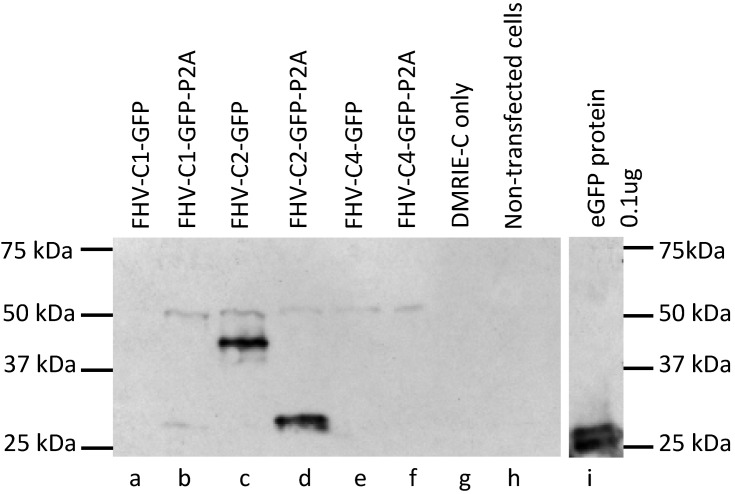
Western blot analysis demonstrating P2A cleavage efficiency in BHK-21 cells at 48 h. Cell lysates were collected at 48 h after confirmation of fluorescence and 20 µL of cell lysate was loaded onto 8%–16% Criterion™ TGX™ precast gel. The gel was transferred to a PVDF blot, and the blot was blocked and incubated with a monoclonal rabbit anti-GFP antibody and secondary incubation was with a goat-HRP-anti-rabbit antibody. Blots were developed with a HRP development kit after an hour and exposed on X-ray film. (**a**) FHV-C1-GFP; (**b**) FHV-C1-GFP-P2A; (**c**) FHV-C2-GFP; (**d**) FHV-C2-GFP-P2A; (**e**) FHV-C4-GFP; (**f**) FHV-C4-GFP-P2A; (**g**) DMRIE-C only; (**h**) un-transfected cells; and (**i**) eGFP protein 0.1 µg.

After confirmation of fluorescence, cell lysates were collected at 48 h from P2A clones and Western blot analysis was performed (Figure 4). Addition of P2A restored GFP expression (lane b) to FHV-C1-GFP (lane a) and was indicative of a successful cleavage reaction. Fusion of the B2 protein (11.6 kDa) and GFP (26.9 kDa) is evident with FHV-C2-GFP (lane c) where GFP is detected at 38.5 kDa instead of 26.9 kDa and successful cleavage of P2A in FHV-C2-GFP-P2A (lane d) clone was observed. Insertion of P2A cleavage sequence in the FHV-GFP clones allowed for the separation of GFP from B2 protein in clone FHV-C2-GFP and prevented a fusion protein translation read through. GFP accumulation was highest for FHV-C2-GFP (lane c) and FHV-C2-GFP-P2A (lane d). GFP protein was undetectable via western analysis with FHV-C1-GFP (lane a), FHV-C4-GFP (lane e) and FHV-C4-GFP-P2A (lane f).

### 2.5. Fluorescence of FHV-C2-OA Clones

**Figure 5:**
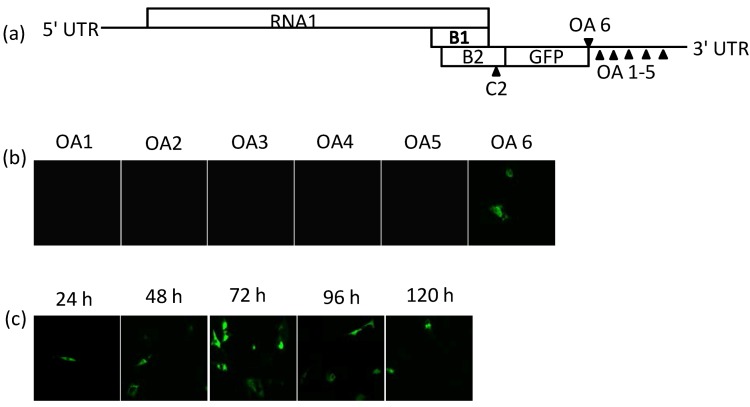
Construction and characterization of FHV-OA clones. (**a**) Schematic diagram showing the 6 OA insertion sites; (**b**) Fluorescence images of FHV-GFP-OA clones that were taken at 48 h after liposome-mediated transfection of BHK-21 cells; and (**c**) Time course assay showing GFP expression kinetics of FHV-C2-GFP-OA in BHK-21 cells after transfection with 2 µg of FHV-C2-GFP-OA RNA. All cell images are at 400× magnification.

As the highest level of GFP accumulation was obtained at the C2 position (Figure 2b, Figure 3a and Figure 4c), TMV OA was introduced into the FHV-C2-GFP clone at the different positions in the 3' UTR (Figure 5a). Fluorescence was not observed with any of the clones where OA was introduced into the 3' UTR (Figure 5b). Expression was observed at the OA6 position, which is directly downstream of GFP (Figure 5b) and this clone, *i.e.*, FHV-C2-GFP-OA, followed the expression pattern of the parental FHV-C2-GFP. Although, GFP expression was reduced in comparison with the parental FHV-C2-GFP clone, fluorescence was observed within 24 h, increased by 72 h and reduced over time (Figure 5c), indicating the replication and subgenomic functions of RNA1 was still intact.

### 2.6. Self-Assembly of Virus-Like Nanoparticles

**Figure 6:**
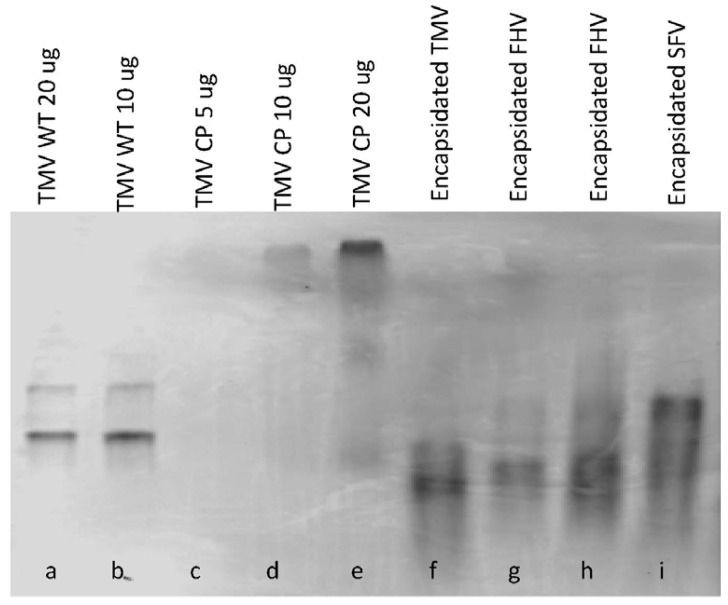
Characterization of *in vitro* encapsidated products by comparing migration rates on a Coomassie-stained 0.5% *w*/*v* agarose gel. TMV CP was mixed with OA-containing RNAs in the presence of a 25 mM phosphate buffer and incubated overnight at room temperature. The resulting mixture was polyethelene glycol (PEG) precipitated, pelleted and resuspended with 50 µL nuclease free PBS. 20 µg of encapsidated product was loaded onto a 0.5% Tris-phosphate-EDTA (TPE) gel and electrophoresis was performed at 300 mA for 3 h. The gel was stained with Coomassie blue overnight and de-stained for 2 days. TMV wild type virus and TMV CP were run as a control and size markers where the TMV WT monomer runs at 6 kb and dimerruns at 12 kb: (**a**) TMV WT 20 µg; (**b**) TMV WT 10 µg; (**c**) TMV CP 5 µg; (**d**) TMV CP 10 µg; (**e**) TMV CP 20 µg; (**f**) *in vitro* encapsidated wt TMV RNA; (**g**/**h**) *in vitro* encapsidated FHV-C2-GFP-OA, two independent encapsidations; and (**i**) *In vitro* encapsidated SFV-β-Gal-OA was also run as a positive control for successful encapsidation.

The FHV-C2-GFP-OA was tested for the ability to support encapsidation. The size difference among the different encapsidation products was demonstrated via an agarose gel (Figure 6). As a control SFV-β-gal-OA RNA was also combined with TMV CP in the presence of a phosphate buffer [5]. Wild-type (WT) TMV ran as a doublet and served as a size marker and is shown as a well-defined intense band of 6 kb and a less intense upper band of about 12 kb that are most likely TMV particles associated end-to-end (lanes a and b). In comparison, TMV CP migrated much more slowly in the gel, a notable difference due to the lack of negatively charged RNA (lanes c–e). In addition to serving as a control and size marker, the TMV CP also functioned as an indicator of complete encapsidation. TMV encapsidated FHV-C2-GFP-OA showed little or no free coat protein, and migrated to a lower position than TMV encapsidated products (lanes f and g), which is expected as FHV-C2-GFP-OA RNA is approximately 3.8 kb in length and encapsidated rod size is directly proportional to the RNA length [2]. In contrast, the 12 kb SFV-β-gal-OA RNA encapsidated particles ran at the approximate location of the TMV doublet (~12 kb), as has been seen before [5].

### 2.7. Electron Microscopy Analysis of Encapsidated Products

**Figure 7:**
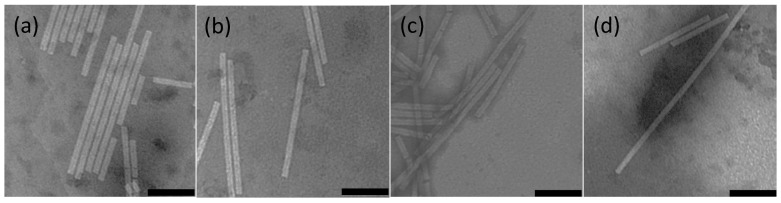
Electron micrographs of *in vitro* encapsidated (IVE) OA-containing RNA with TMV CP. Grids were coated with 100–200 µg/mL of *in vitro* encapsidated particles, negatively stained with 1% phosphotungstic acid (PTA), air dried, observed with a Philips CM120 microscope and imaged with Gatan MegaScan 795 digital camera. Black bar represents 100 nm: (**a**) Wild type TMV virions approximately 300 nm in length; (**b**) TMV RNA encapsidated with TMV coat proteins are approximately 300 nm in length; (**c**) FHV-C2-GFP-OA RNA encapsidated with TMV coat proteins approximately 187nm in length; and (**d**) SFV-GFP-OA RNA encapsidated with TMV coat proteins approximately 600nm in length.

The resulting *in vitro* encapsidated products were visualized by electron microscopy to determine if TMV-like rods formed after encapsidation. *In vitro* encapsidated wild type TMV RNA (Figure 7b) was visually indistinguishable from wild type TMV (Figure 7a) via electron microscopy; while rods of variable lengths were present, rods of the expected length of 300 nm were more prevalent. When FHV-C2-GFP-OA RNA was mixed with wild type TMV CP, the resulting monomer rods were proportional to FHV-RNA length compared to *in vitro* encapsidated TMV (3.8 kb FHV RNA *vs.* 6.1 kb wild type TMV RNA, or 62% of 300 nm, for an expected length of 187 nm) and measured 187 ± 7.6 nm in length (Figure 7d). A previously created replicon, SFV-β-gal-OA, was also tested as a control, and as expected, the rods were approximately double in length of TMV encapsidated rods (Figure 7c) which correlates with previous observations [5].

### 2.8. Discussion

We have used the self-assembly properties of TMV coat protein, and modified FHV with the TMV OA, to direct the formation of a novel nanoparticle composition. Our studies show that TMV coat protein self-assembly can override native packaging signals typical of Nodaviridae family members to allow for specific encapsidation and packaging of the viral genome by a heterologous structural protein. In studies by Annamalai *et al.*, the native FHV genome packaging specificity was determined to be dependent on replication and translation of the FHV CP at the mitochondrial membrane of infected cells, which is better known as replication-coupled packaging [7]. Our data demonstrated that by introducing the TMV OA packaging signal, the native packaging of FHV RNA was uncoupled, while FHV RNA1 functionality and transgene expression capacity was maintained as indicated by maintenance of GFP expression. This extends our previous observations with Semliki Forest virus (SFV)-β-Gal-OA encapsidations [5] to a structurally different class of virus RNA. Because of the advantages of FHV over SFV, especially small genome size, we will now be able to more fully explore the uses of nodamuravirus encapsidated RNA, either in synthetic or *in vivo* encapsidation studies.

Due to the small nature of the FHV genome, we found that the number of possible GFP insertion sites was limited to the 3' end of RNA1 in either the B1or B2 ORF. The best FHV insertion site was determined to be downstream of the RdRp, as a fusion of GFP to the B2 protein, FHV-C2-GFP which correlates with previous studies [23]. One novel method to improve transgene expression in the low expressing clones, FHV-C1-GFP and FHV-C4-GFP, was to use a protease cleavage site, P2A, to disassociate GFP from host FHV protein fusions. C1 is located directly before the stop codon for protein A or the end of the B1 ORF and protein expression at this position is largely driven by RNA1 replication unlike the other 3 clones which are driven by RNA3 accumulation and translation [23]. Significant improvements in GFP expression was observed with the addition of P2A upstream of the C1 position which separated GFP from the 3' terminus of protein A/B1 suggesting that fusion proteins at this position were not well tolerated. The C4 insertion site that was created at the 3' termini of the B2 protein created a B2-GFP fusion protein and resulted in weak to little fluorescence. Previous studies have shown that fusion with B2 protein does not inhibit initial cycle of RNA1 replication in mammalian cells, however, RNA replication is inhibited through serial passaging which could explain the weaker GFP expression at the C4 position [29].

In an effort to improve GFP expression at the C4 position, two approaches were investigated. P2A cleavage sequence was engineered into the C4 clone but did not improve GFP expression. In the second approach, two different insertions of the 3' termini of RNA1, ds1 and ds2, were also tested to improve C4 GFP expression. The ds1 and ds2 regions are duplicates of the FHV RNA 3 region thus allowing for the manipulation of upstream sub-genomic promoters while sustaining B2 ORF translation and function [6]. These regions have been identified in the FHV genome as cis acting elements that may assist in RNA1 replication [6]. Improved expression of GFP was also observed with the addition of nucleotides 509–868 from RNA2 into the FHV-C4-GFP clone, which indicated that the 509–868 bp region of FHV RNA2 could function as an independent sub-genomic promoter. This is in line with a study describing a region ranging from 517–728 bp that was necessary for nodavirus RNA2 replication [26]. In another deletion mutant study, the region between 525 and 725 was determined to be important for the maintenance of RNA2 replication and a RNA2 deletion mutant, DI 634, was discovered where only 634 bp of the 1400 bp RNA2 was enough to maintain replication [30]. The cDNA clone of DI634 was then used to drive GFP translation and expression in Drosophila cells and in mosquitoes; however, the GFP insertion was at position 58 of DI634 and not further downstream [31]. Our data supports these previous observations, and further suggests that this region of FHV RNA2 can also be used to drive transgene expression and can function independently as a subgenomic promoter.

Variations of *in vitro* self-assembly of TMV CP and TMV RNA have been studied extensively [1,2]. TMV-like particles have been created in *Escherichia coli* when TMV CP and OA-containing RNAs were co-expressed [32] and in transgenic tobacco plants where stably expressed OA-containing RNA was encapsidated in a TMV infected plant [4,33]. In Schizosaccharomyces pombe, expression of TMV CP was able to encapsidate non-OA containing RNAs as well, resulting in TMV-like rods that were heterogeneous in length [34]. However, including the OA in the RNA scaffolds resulted in TMV-like rods proportional in length to the OA-RNAs [34]. In our previous studies of TMV CP transencapsidation by self-assembly, we utilized an SFV genomic RNA backbone and demonstrated maintenance of RNA function, and expression of transgenes *in vivo* and *in vitro*. SFV is an alphavirus, distantly related to TMV, but of relatively similar genomic organization and subgenomic RNA utilization. As FHV utilizes replication-coupled packaging, and long distance pairing to support native subgenomic RNA replication [6], it was not clear at the outset that FHV could successfully be modified for encapsidation by TMV CP. Clearly, not all sites of OA insertion were tolerated, as measured by sustained FHV GFP expression, suggesting there are limitations to modifications in FHV RNA sequence, structure, or both. However, our work clearly demonstrates that FHV can be packaged into nanoparticles independently of replication, using the TMV CP self-assembly process.

Future studies will expand the use of encapsidated FHV as a vaccine. It has been recently demonstrated that TMV interacts directly with antigen presenting cells [35], and that TMV can provide adjuvant effects sufficient to protect mice against pathogen challenge [36]. We want to further enhance these properties by delivery of a functional RNA capable of stimulating immunity to a target antigen. Our goal is to further optimize the encapsidation process, by testing the ability of *in planta* expressed FHV-GFP-OA RNA to interact with TMV coat protein *in vivo*, to overcome *in vitro* RNA synthesis costs, and capping limitations, and deliver RNA that will have more robust capacity for co-translational disassembly. We feel that the continued study of macromolecular assembly of functional RNA by TMV coat protein will extend the use of encapsidation, improve expression of target genes for infectious disease and cancer, and allow for the development of new cost effective vaccines.

## 3. Experimental Section

### 3.1. Cells

Baby hamster kidney cells (BHK-21) were maintained in modified Eagle’s media (MEM-Invitrogen, Carlsbad, CA, USA) supplemented with 10% heat-inactivated fetal bovine serum (Invitrogen, Carlsbad, CA, USA) and 5% penicillin/streptomycin mixture (Invitrogen, Carlsbad, CA, USA).

### 3.2. Plasmid Construction

Standard molecular cloning techniques were used unless otherwise stated. The plasmid containing the FHV RNA1 plasmid (FHV [1,0]) that was kindly provided to us by Dr. Andrew Ball, is a T7 promoter driven plasmid and is described in detail elsewhere [29]. Four insertion sites, C1, 3034 bp; C2, 3037 bp; C3, 2731 bp; C4, 3055 bp, were created for the introduction of the reporter gene, eGFP. A polylinker, CTCGAGGCGATCGCCTGCAG, encompassing three restriction sites, XhoI, AsiSI and PstI was cloned into positions C1–C4 in the FHV RNA1 plasmid (Figure 1a). After confirmation by sequencing and restriction digest analysis, eGFP was introduced into these sites to create FHV-C(1–4)-GFP clones. To further improve GFP expression and allow for cleavage of the eGFP protein in the fusion clones FHV-C1, C2, C4-GFP, the P2A self-cleavage sequence, GGAAGCGGAGCTACTAACTTCAGCCTGCTGAAGCAGGCTGGA GACGTGGAGGAGAACCCTGGACCT, from Porcine Teschovirus-1 [37] was introduced into positions FHV-C1, C2, C4 directly upstream of the eGFP gene to generate FHV-C1, C2, C4-GFP-P2A clones (Figure 1b). The P2A sequence was not added to FHV-C3-GFP as it would allow for the cleavage of the 3' end of the polymerase gene leading to a truncated protein A with no functionality. Additional expression clones were generated where the sub-genomic regions from the 3' end of FHV RNA1, 2518–3055 and 2518–3107 bp respectively, were amplified and cloned into site C4 for improvement of GFP expression (Figure 1c) [6]. As the entire RNA3 region of FHV RNA1 was duplicated, these clones are called double sub-genomics (ds) and labeled FHV-C4-GFP-ds1 and FHV-C4-GFP-ds2 respectively [6]. Another strategy to improve GFP expression at the C4 position was to utilize the subgenomic promoter-like region from RNA2 [16]. The FHV RNA2 plasmid was kindly provided to us by Dr. Anette Schneemann and was used to amplify genomic fragment 509–868 from RNA2 and was cloned into the FHV-C4-GFP vector to create FHV-C4-GFP-RNA2 (Figure 1d). The origin of assembly (OA) from TMV was introduced into five insertion sites in the RNA1 3' UTR, *i.e.*, OA1, 3062 bp, OA2 3068 bp, OA3 3078 bp, OA4 3090 bp and OA5 3100 bp (Figure 5a). These genomic positions were selected to minimize disruption of predicted secondary RNA structures. The OA was also inserted downstream of GFP in the PstI site of C2 to create OA 6 clone (Figure 5a). All clones were sequenced to ensure maintenance of sequence identity and to assess for spurious mutations in the clones.

### 3.3. RNA Transcription and Transfection

Full length capped RNA1 transcripts were generated *in vitro* via a T7 promoter driven kit (mMESSAGE mMACHINETM, Ambion, TX, USA). Briefly, 1 µg of plasmid DNA was mixed with kit contents and incubated at 37 °C for 2 h. Transcribed RNA was purified using Qiagen RNeasy Mini Kit (Germantown, MD, USA), quantified by UV spectrometry (Nanodrop, Wilmington, DE, USA) and stored at −80 °C until further use. BHK-21 cells were grown to confluency in a 12-well plate. Cells were washed twice with PBS, 500 µL of Opti-MEM media was mixed with 2 µg of FHV RNA1 transcripts and 6 µL of DMRIE-C and the transfection mixture was added to cells. Cells were incubated at 37 °C for 4 h after which transfection media was replaced with fresh cell growth media and the plate was moved to a 28 °C incubator and incubated for 120 h. A Semliki Forest virus (SFV) GFP expression construct was used as a control for positive GFP expression. FHV RNA1 was used as a negative transfection control.

### 3.4. GFP Imaging by Fluorescence Microscopy and Western Blot Analysis

BHK-21cells were observed for GFP expression daily for 120 h post-transfection using a Nikon Eclipse TS-100 microscope (Melville, NY, USA) and NIS Elements BR 4.11.00 imaging software (Nikon, Melville, NY, USA) and compared to SFV-GFP expression vector as a control. After confirmation of GFP expression by imaging, cells were lysed with SDS-PAGE buffer, lysates collected and boiled for 5 min at 98 °C. Lysates were loaded on 8%–16% Criterion™ TGX™ precast gels (Bio-Rad Labs, Hercules, CA, USA) and transferred to PDVF membranes (Biorad, Hercules, CA, USA) by semi-dry transfer according to standard protocols. Blots were blocked, incubated with a 1:2000 dilution of rabbit anti-GFP antibody (Polysciences, Warrington, PA, USA), washed and then incubated with a 1:2000 goat anti-rabbit antibody (Pierce, Rockford, IL, USA). After 1 h, membranes were developed using HRP development kit (Pierce) and visualized by X-ray film (Pierce, Rockford, IL, USA).

### 3.5. Purification of TMV and TMV Coat Protein (CP)

TMV was isolated from infected plant tissue as previously described [1,2]. The final virus preparation was re-clarified by centrifugation at 18,000 rpm for 30 min at 4 °C. To ensure that the TMV prep was RNase free, RNase activity tests were carried out by incubating 2 µg of control TMV RNA with a serial dilution of purified TMV at 37 °C for 2 h. The resulting reactions were run out on agarose gels to confirm the absence of RNase activity. A standard bicinchoninic acid assay (BCA; Pierce, Rockford, IL, USA) assay was used for quantification of purified TMV, and 8%–16% SDS-PAGE analysis (Biorad, Hercules, CA, USA) was performed to confirm TMV purity. Purification of TMV coat protein was performed as previously described [5]. Briefly, 1 volume of cold, purified TMV was mixed with 2 volumes of glacial acetic acid and incubated on ice for 30 min. After confirmation of precipitation, the reaction was centrifuged for 30 min at 4 °C at 18,000 rpm. Supernatant was decanted, and sample was re-centrifuged to ensure that all viral RNA was pelleted. The supernatant was diluted with an equal volume of cold water and dialyzed (Snakeskin 6000–8000 MWCO, Pierce) against cold water. After 3 days, when the TMV coat protein reached its isoelectric point a white precipitate was aggregated. Dialyzed sample was centrifuged again at 18,000 rpm for 30 min at 4 °C. Pelleted coat protein was re-suspended in RNase free water, dissolved with the addition of sodium hydroxide to pH 7.0, and centrifuged at 18,000 rpm for 30 min at 4 °C to remove the aggregated coat. The supernatant was removed and stored at 4 °C with 0.02% sodium azide. A BCA assay and RNase activity test was performed on the final coat prep to ensure lack of RNase activity and quantify TMV coat protein (CP) recovery.

### 3.6. Assembly of Nanoparticles

TMV CP was mixed with 50 mM of phosphate buffer and incubated at room temperature (RT) overnight to form assembly competent discs. Phosphate-treated CP was centrifuged at 10,000× *g* for 10 min at 4 °C. Supernatant was removed and CP recovery was quantified using a BCA assay. *In vitro* encapsidation was set up with 300 µg of phosphate-treated TMV CP and 15 µg of purified RNA in the presence of a 25 mM phosphate buffer and incubated overnight at room temperature. The encapsidation reaction was mixed with polyethylene glycol (PEG) and incubated for 1 h on ice after which the reaction was centrifuged at 14,000× *g* for 10 min at 4 °C. Supernatant was removed and the resulting pellet was resuspended in 50 µL of sterile 1× PBS (Invitrogen, Carlsbad, CA, USA). Agarose gel electrophoresis and electron microscopy was used to visualize FHV-TMV-like nanoparticles as previously described [5].

### 3.7. Electron Microscopy

Grids (400 Mesh copper, carbon coated; Ted Pella, Bedding, CA, USA) were floated on drops of encapsidated samples and diluted to a concentration of 100–200 µg/mL in 0.1 M phosphate buffer, pH 7.0. After 15 min of contact time, excess liquid was removed, the grids negatively stained with 1% phosphotungstic acid (PTA) and air dried. All samples were observed with a Philips CM120 microscope that was coupled to a Gatan MegaScan 795 digital camera (Pleasanton, CA, USA).

## 4. Conclusions

We have shown successful self-assembly of TMV CP onto an OA modified FHV RNA genome. Since FHV is able to replicate in plants, co-expression of FHV RNA and TMV coat protein is feasible, making the FHV-TMV system promising in terms of future vaccine development [12,13]. One of the advantages of utilizing plant viral coat proteins such as TMV CP is that TMV-like particles can present antigens directly to dendritic cells and other antigen presenting cells due to their size and nanoparticulate nature making them useful in a vaccine setting [35,38]. TMV nanoparticle vaccines avoid immune surveillance and sustain repeat boosting, unlike most native viruses, which are subject to aggressive antibody neutralization of virus capsid after a single dose. Further studies testing assembly of FHV nanoparticles *in planta*, and *in vivo* animal studies testing vaccine efficacy, will enhance characterization of the FHV-TMV expression system and demonstrate utility in delivery of antigenic epitopes of medical importance.
